# Psychological resilience and music performance anxiety: exploring mediators and sex differences in Chinese choir members

**DOI:** 10.3389/fpsyg.2025.1703571

**Published:** 2025-10-21

**Authors:** Hao Du, Yanchang Liu, Jian Sun

**Affiliations:** ^1^Xichang University, Xichang, China; ^2^Guangxi Minzu University, Nanning, China; ^3^Xihua University, Chengdu, China

**Keywords:** psychological resilience, music performance anxiety, Chinese choirs, sex differences, performance control sense, pre-performance rumination

## Abstract

Music performance anxiety (MPA) is a prevalent psychological challenge that can impair musicians' wellbeing and performance quality. While psychological resilience has been proposed as a protective factor, the mechanisms underlying its association with MPA remain unclear, particularly among choir members. This study examined the direct and indirect relationships between psychological resilience and MPA via performance control sense and pre-performance rumination, and further explored whether these pathways differed by sex. A total of 801 Chinese choir members completed validated measures of psychological resilience, performance control sense, pre-performance rumination, and MPA. Structural equation modeling (SEM) was conducted to test mediation effects, and multi-group SEM was used to assess sex differences. Results showed that resilience was negatively associated with MPA both directly and indirectly. Performance control sense emerged as the dominant mediator, accounting for 53.8% of the total effect, while pre-performance rumination accounted for 7.7%. Multi-group analyses indicated significant sex differences: resilience was more strongly linked to reduced rumination in females, the protective effect of control beliefs was stronger among females, and the maladaptive impact of rumination was stronger among males. These findings highlight psychological resilience as a key protective factor in MPA, primarily by enhancing control beliefs and reducing rumination. The study contributes to theoretical understanding of sex-specific mechanisms and suggests practical implications for resilience-based, sex-sensitive interventions in music education and choir practice.

## 1 Introduction

In the fast-paced and high-pressure modern society, music performance anxiety (MPA) has become a widespread concern, particularly in the realm of artistic performance (Papageorgi, 2020). MPA is a common phenomenon characterized by a range of negative physiological, cognitive, and behavioral symptoms occurring before or during a stage performance, such as palpitations, sweating, difficulty concentrating, muscle tension, and avoidance of performance (Lima et al., 2024). MPA not only severely impacts performers' artistic expression but can also have long-term negative effects on their mental health, hindering their career development and artistic potential (Gómez-López and Sánchez-Cabrero, 2023; Kinney et al., 2025). In China, with the popularization of art education and the flourishing of cultural undertakings, the number of individuals involved in music performance is increasing (Wang and Yang, 2024). Choral singing, as a collective art form, presents its members with MPA issues that also warrant attention. However, research specifically targeting MPA among Chinese choir members is relatively scarce, and the exploration of its influencing factors and underlying mechanisms remains insufficiently in-depth.

Psychological resilience, as a positive psychological quality, is defined as an individual's ability to successfully adapt and return to normal functioning in the face of adversity, trauma, tragedy, threats, or other significant sources of stress (Connor and Davidson, 2003; Lu et al., 2025). Individuals with high psychological resilience tend to cope more effectively with stress, recover quickly from negative emotions, and maintain a positive and optimistic outlook (Booth and Neill, 2017; Lu et al., 2024b). In recent years, an increasing number of studies have begun to focus on the role of psychological resilience in coping with various psychological stresses, including MPA (Yang et al., 2025). For music performers, possessing higher psychological resilience may help them better manage performance pressure and mitigate the negative impact of MPA.

The ways in which psychological resilience influences MPA are multifaceted. Firstly, individuals possessing high psychological resilience often exhibit a stronger sense of self-efficacy and self-confidence (Leng et al., 2025). This helps them maintain a positive self-perception in performance situations and reduces worries about potential failure. Secondly, resilient individuals are more inclined to actively seek and utilize resources from others or their environment to alleviate anxiety (Rudenstine et al., 2025). Numerous studies have demonstrated that high psychological resilience can effectively buffer the negative impact of various stressors on an individual's mental health (Ayed et al., 2025; Baminiwatta et al., 2025). Specifically, within the performance domain, research has found that performers with higher psychological resilience are better able to employ positive coping strategies, such as positive cognitive reappraisal and problem-solving, when faced with performance pressure, thereby reducing their experiences of anxiety (Yang et al., 2025). For instance, studies of classical musicians and music students revealed that individuals with higher levels of psychological resilience reported significantly lower anxiety levels (Kegelaers et al., 2021; Yang et al., 2025). This suggests that psychological resilience may protect performers from anxiety by enhancing their psychological adaptability and stress coping mechanisms. Consequently, this study hypothesizes that psychological resilience is negatively correlated with MPA, meaning that choir members with higher psychological resilience will experience lower levels of MPA.

Exploring the mediation mechanisms between psychological resilience and MPA is crucial for developing effective intervention strategies. Performance control sense, as an important cognitive factor, refers to an individual's perceived degree of control over performance outcomes or processes in a performance situation (Osborne and Franklin, 2002). This mediating mechanism can be elucidated primarily through the lens of Social Cognitive Theory (Bandura, 2001), which posits that personal factors (e.g., beliefs, self-efficacy), behavior, and the environment dynamically interact. Resilient individuals often demonstrate an internal locus of control, high self-efficacy, and strong mastery expectations—beliefs that reduce threat perception and support adaptive performance behavior (Maddux and Kleiman, 2021). These individuals tend to appraise performance situations as manageable challenges, which diminishes anticipatory anxiety and enhances focused attention (Zhang and Jenatabadi, 2024). These control beliefs minimize the perceived threat and uncertainty inherent in performing before an audience, shifting attention away from potential failures and toward proactive management and successful execution (Albaqawi et al., 2025). In contrast, individuals with low performance control sense are more likely to catastrophize, experience fear of negative evaluation, and engage in avoidance or safety behaviors that exacerbate anxiety symptoms (Zeng et al., 2021). Particularly in musical contexts, where external scrutiny is intense, the absence of perceived control can intensify physiological arousal and cognitive interference. Therefore, performance control sense may serve as a mediator: it shapes how performers interpret stress and determines whether psychological resilience translates into effective coping or heightened MPA.

Empirical research may support the role of performance control sense as a key mediator in the resilience–MPA relationship. Individuals with higher psychological resilience tend to report a stronger sense of control in demanding performance situations and are more likely to employ problem-focused coping strategies, which are associated with reduced anxiety and improved engagement with the task at hand (Li and Zheng, 2025). These strategies promote mastery experiences and reduce the likelihood of threat-based interpretations of performance challenges. In the musical context, low performance control has been consistently linked to elevated MPA. Musicians who perceive limited control over performance outcomes often experience more anxiety symptoms, particularly in public or evaluative settings (Guyon et al., 2022). Furthermore, distorted control beliefs have been found to predict maladaptive thought patterns and increased physiological arousal during performances (Moral-Bofill et al., 2022). Such individuals are more likely to feel overwhelmed, doubt their abilities, and engage in negative self-appraisal, which further reinforces MPA. Based on this evidence, the present study hypothesizes that performance control sense mediates the impact of psychological resilience on MPA. Specifically, choir members with greater psychological resilience are expected to demonstrate a stronger belief in their capacity to manage performance demands, thereby experiencing lower levels of anxiety.

In addition to performance control sense, pre-performance rumination may also be a critical mediating factor linking psychological resilience and MPA. Rumination is a repetitive, passive thinking pattern focused on one's distress, its possible causes, and consequences (Watkins and Roberts, 2020). In a performance context, pre-performance rumination refers to an individual's repeated thoughts about negative events related to the performance, worrying about potential mistakes, excessively focusing on their physiological reactions, or catastrophizing about performance outcomes before taking the stage (Kaleńska-Rodzaj, 2021). This thinking pattern often consumes cognitive resources, exacerbates negative emotions, and hinders the adoption of active and effective coping strategies (Kaleńska-Rodzaj, 2021).

According to Gross's process model of emotion regulation (Gross and Thompson, 2007), the way individuals regulate their emotions is shaped by the timing and strategies they employ in response to emotionally salient situations. Psychological resilience is associated with the use of adaptive antecedent-focused strategies, such as cognitive reappraisal, which occur early in the emotion generative process (Yang et al., 2025). By reframing performance situations as manageable challenges rather than potential threats, resilient individuals are less likely to engage in maladaptive response-focused strategies such as pre-performance rumination (Yu and Liu, 2024). Rumination represents a perseverative focus on possible negative outcomes and one's own inadequacies, which amplifies negative affect and undermines effective emotion regulation (Watkins and Roberts, 2020). Consequently, lower levels of psychological resilience may predispose individuals to heightened rumination before a performance, which in turn intensifies MPA. Thus, the mediating role of pre-performance rumination can be explained by the process model: high psychological resilience facilitates early adaptive regulation that reduces ruminative tendencies, whereas low psychological resilience fosters maladaptive rumination that exacerbates MPA.

Empirical evidence increasingly indicates that psychological resilience serves as a crucial protective factor against ruminative thinking. Large-scale studies have demonstrated that psychological resilience significantly and negatively predicts cognitive rumination (Mulawarman et al., 2024). Consistent patterns have been observed across diverse populations, including adolescents, cancer survivors, and individuals with chronic illnesses, suggesting a broad effect of psychological resilience in mitigating ruminative tendencies (Chang et al., 2023; Liu et al., 2023; Ping Chuang et al., 2024). Mechanistically, resilient individuals are more likely to employ adaptive regulatory strategies, such as cognitive reappraisal and problem-solving, rather than engaging in maladaptive rumination (Yang et al., 2025). However, to date, no empirical research has directly examined the pathway from psychological resilience to pre-performance rumination. Within the domain of music performance, excessive pre-performance rumination, particularly concerning anticipated failure or somatic symptoms, has been consistently associated with elevated MPA (Kaleńska-Rodzaj, 2019, 2021). Integrating these lines of evidence, it is plausible to hypothesize that psychological resilience may indirectly alleviate MPA by reducing pre-performance rumination. Accordingly, choir members exhibiting higher psychological resilience are expected to engage in less anticipatory rumination, thereby experiencing lower levels of MPA.

Sex differences are an important and frequently explored variable in psychological research, particularly concerning emotional distress and stress coping. Accumulated evidence has consistently demonstrated that males and females differ in reported anxiety levels, emotion regulation strategies, and coping styles when confronted with stressful situations (McLean et al., 2011; Johnson and Whisman, 2013). Within the context of MPA, studies have frequently reported that female performers tend to experience and report higher levels of MPA compared to their male counterparts (Cui et al., 2024). Regarding psychological resilience, some studies indicate that males and females may differ in certain dimensions of psychological resilience (Boardman et al., 2008; Sardella et al., 2022). These differences may manifest in the choice of coping strategies; for instance, females might be more inclined to seek social support, while men might prefer to solve problems independently. Furthermore, performance control sense and pre-performance rumination may also exhibit sex differences. For example, females might be more susceptible to social evaluation, thereby reducing their performance control sense and increasing their tendency for pre-performance rumination (Johnson and Whisman, 2013).

Beyond individual constructs, potential sex differences may also influence the relationships between these psychological variables. For example, the association between psychological resilience and pre-performance rumination may be moderated by sex. Previous research suggests that females are generally more prone to ruminative coping when experiencing stress, whereas males are more likely to utilize avoidance or behavioral distraction techniques (Johnson and Whisman, 2013). Consequently, psychological resilience may be less effective in mitigating rumination for females than for males. Similarly, the pathway from rumination to MPA may differ across sexes, as females, due to their greater emotional awareness and expressivity, might be more vulnerable to the distress-amplifying effects of sustained rumination (Stuart et al., 2025). Although empirical investigations directly testing these moderation effects remain limited, theoretical frameworks on sex differences in emotional processing lend support to their plausibility. Furthermore, the mediating role of performance control sense in the relationship between psychological resilience and MPA may operate in a sex-specific manner. For females, elevated psychological resilience may not necessarily result in a strong sense of control under evaluative pressure, possibly due to a heightened sensitivity to negative feedback or social comparison (Wang et al., 2023). In contrast, males may derive a greater sense of control from psychological resilience, which could lead to lower anxiety in performance settings (Wang et al., 2023). Thus, the present study hypothesizes that the direct and indirect relationships between psychological resilience and MPA may differ by sex.

**Figure 1:**
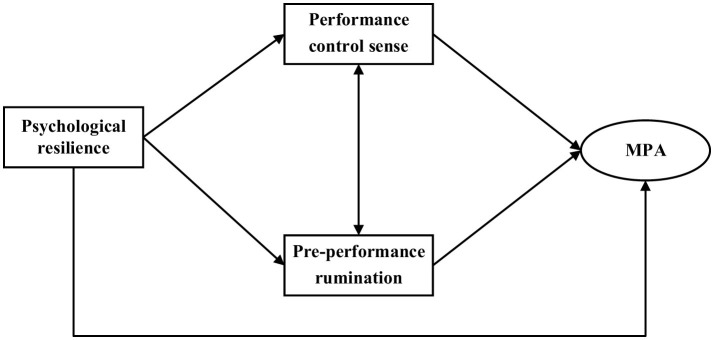
The hypothesized mediation model, MPA, music performance anxiety.

In light of the aforementioned background and theoretical inferences, this study aims to investigate the relationship between psychological resilience and MPA among Chinese choir members, with a particular focus on the mediating roles of performance control sense and pre-performance rumination, while also exploring potential sex differences in these relationships. This study will construct an integrated model to test the following hypotheses: (H1) Psychological resilience is negatively associated with MPA. (H2) Performance control sense would mediate the relationship between psychological resilience and MPA, i.e., higher psychological resilience would enhance performance control sense, thereby reducing MPA. (H3) Pre-performance rumination would mediate the relationship between psychological resilience and MPA, i.e., higher psychological resilience would reduce pre-performance rumination, thereby reducing MPA. (H4) The aforementioned associations may differ between male and female choir members. The findings of this study will not only enrich our understanding of the mechanisms influencing MPA, particularly its manifestation among Chinese choir members, but also provide empirical evidence for developing effective psychological interventions to improve the mental health of music performers. Figure 1 demonstrates the hypothetical model.

## 2 Materials and methods

### 2.1 Participants and data collection

Participants were recruited from 5 community-based choirs located in Sichuan, China. Data collection was conducted between July and August 2025 using Wenjuanxing (www.wjx.cn), a widely used online survey platform in China. Eligible participants were required to be active choir members aged 18 years or older who had taken part in at least one public performance within the past year. Individuals with self-reported severe mental illness or cognitive impairment were excluded. All participants completed the questionnaire voluntarily and anonymously, and electronic informed consent was obtained prior to participation. The study protocol was reviewed and approved by the Ethics Committee of Xihua University (Approval number: XH250715-01).

A total of 819 participants initially completed the questionnaire. Data quality was ensured by excluding cases that failed logical checks (e.g., inconsistencies between item responses; *n* = 18). The final analytic sample comprised 801 respondents, which constituted the basis for subsequent analyses.

### 2.2 Measures

#### 2.2.1 Demographic variables

Demographic variables were collected including age, sex, self-reported family financial situations, employment status, whether having a partner, and whether being a minority.

#### 2.2.2 MPA

MPA was measured using the Chinese version Stage Music Performance Anxiety Inventory (Su et al., 2017). It captures anxiety experienced during actual stage performance and consists of two dimensions: physiological anxiety (e.g., “I feel nervous”) and performance-related worry (e.g., “I worry that today's performance may go wrong”). It includes 14 items rated on a 7-point Likert scale ranging from 1 (never) to 7 (always), with higher scores indicating greater levels of MPA. In the present study, the Cronbach's alpha was 0.95.

#### 2.2.3 Psychological resilience

Psychological resilience was measured using the 10-item short version of the Connor–Davidson Resilience Scale (CD-RISC-10; Connor and Davidson, 2003). The scale assesses an individual's ability to cope with adversity and adapt positively to challenges, such as change, stress, illness, or failure. Each item is rated on a 5-point Likert scale ranging from 0 (not true at all) to 4 (true nearly all of the time), with total scores ranging from 0 to 40. Higher scores indicate greater psychological resilience. The Chinese version of the CD-RISC-10 has demonstrated good psychometric properties among Chinese populations (Lu et al., 2024a). In the present study, the scale demonstrated excellent internal consistency, with a Cronbach's alpha of 0.93.

#### 2.2.4 Performance control sense

Performance control sense was measured using the Controllability subscales of the Kenny Music Performance Anxiety Inventory (Kenny, 2023). The items capture a sense of control and confidence in performance situations (e.g., “I often feel that I cannot control my life”; “Before a concert, I am uncertain about whether I will perform well”). Each item was rated on a 7-point Likert scale 1 (Never) to 7 (Always). The items were reversed, with higher scores indicating higher perceived control. In the present study, the scale demonstrated excellent internal consistency, with a Cronbach's alpha of 0.76.

#### 2.2.5 Pre-performance rumination

Pre-performance rumination was assessed with 2 items adapted from the Revised Kenny Music Performance Anxiety Inventory (Kenny, 2009). The items reflect repetitive negative thoughts and preoccupation prior to performance (e.g., “Before a performance, I replay the event over and over in my mind”; “Before a performance, I worry so much that I cannot sleep”). Responses were rated on a 7-point Likert scale ranging from 1 (Never) to 7 (Always), with higher scores indicating greater levels of rumination before performance. In the present study, the scale demonstrated excellent internal consistency, with a Cronbach's alpha of 0.79.

### 2.3 Data analysis

Descriptive analyses were first conducted for demographic variables. Simple linear regression was used to examine the associations between demographic variables and MPA. Pearson correlation coefficients were then calculated to assess bivariate relationships among key study variables, including MPA, psychological resilience, performance control sense, pre-performance rumination, self-scrutiny, and other scrutiny.

To test the hypothesized mediation effects, Structural Equation Modeling (SEM) with a Maximum Likelihood (ML) estimator was conducted. MPA was constructed as a latent variable, while other variables were observed variables. To further examine whether the hypothesized mediation pathways differed by sex, multi-group SEM analyses were performed across male and female participants. Specifically, the fit of a model in which all structural paths were freely estimated across groups was compared to the fit of a model in which the structural paths were constrained to be equal across groups. A statistically significant chi-square difference between the two models would indicate that the mediation pathways differed by sex (Kline, 2016). Satisfactory model fit indices included Comparative Fit Index (CFI ≥ 0.90), Tucker-Lewis Index (TLI ≥ 0.90), Root Mean Square Error of Approximation (RMSEA ≤ 0.08), and Standardized Root Mean Square Residual (SRMR ≤ 0.08) (Little, 2013). All SEM analyses were tested using Mplus 7.3 and other analyses were conducted with SPSS 26.0. Statistical significance was defined as two-tailed *p*-value < 0.05.

## 3 Results

### 3.1 Descriptive statistics

As presented in Table 1, the majority of participants were female (73.4%). Most respondents reported currently having a partner (69.7%) and holding a college degree or above (82.3%). Regarding socioeconomic status, 21.5% self-reported a monthly personal income of less than 4,000 Yuan, whereas 12.0% reported a household income exceeding 10,000 Yuan. In terms of occupational status, 27.1% of the participants were employed full time, and 20.8% were students.

**Table 1:** . Participants' characteristics and associations with MPA.

**Variables**	***n***	**%**	**β**
Age	N.A	N.A	−0.23^***^
**Sex**			
Female	213	26.6	Ref
Male	588	73.4	−0.17^***^
**Educational level**			
Middle school or below	22	2.7	Ref
High school	120	15.0	−0.07
Three-year college	142	17.7	−0.06
University	425	53.1	−0.02
Master or above	92	11.5	−0.05
**Self-reported household income per month**			
< 4,000 Yuan	172	21.5	Ref
4,000–5,999 Yuan	129	16.1	−0.15^**^
6,000–7,999 Yuan	96	12.0	−0.16^***^
8,000–9,999 Yuan	58	7.2	−0.13^***^
>10,000 Yuan	96	12.0	−0.21^***^
Choosing not to report	250	31.2	−0.14^**^
**Employment status**			
Students	167	20.8	Ref
Full-time	217	27.1	−0.22^***^
Unemployed	36	4.5	−0.17^***^
Choosing not to report	381	47.6	−0.16^***^
**Whether having a partner**			
Yes	558	69.7	Ref
No	243	30.3	0.07

^**^p < 0.01.

^***^p < 0.001.

N.A, not applicable; MPA, music performance anxiety; Ref., reference group.

The descriptive statistics for the key study variables are summarized in Table 2. The mean age of participants was 47.74 years, with a range of 22 to 64 years. The mean (SD) score for psychological resilience was 27.05 (6.78), with values ranging from 0 to 40. The mean (SD) performance control score was 10.25 (6.24), with a range of 2 to 14. For pre-performance rumination, the mean (SD) score was 6.85 (2.62). The mean (SD) score for MPA was 40.15 (16.08).

**Table 2:** Participants' characteristics for continuous variables.

**Variables**	**Range**	**Mean**	**SD**
Age	22–64	47.74	18.69
Resilience	0–40	27.05	6.78
Performance control sense	2–14	10.25	2.64
Pre-performance rumination	2–14	6.85	2.62
MPA	14–98	40.15	16.08

MPA, music performance anxiety.

### 3.2 The associations between demographic variables and MPA

Age (β = –0.27), sex (reference group = male, female: β = –0.17), self-reported household income per month (reference group = < 4,000 Yuan, 4,000–5,999 Yuan: β = –0.15; 6,000–7,999 Yuan: β = −0.16; 8,000–9,999 Yuan: β = −0.13; > 10,000 Yuan: β = −0.21), and employment status (reference group = students, full-time: β = −0.22; unemployed: β = −0.17) were negatively associated with MPA. In contrast, educational level and whether having a partner were not significantly associated with MPA (Table 1).

### 3.3 Pearson correlations

As presented in Table 3, psychological resilience demonstrated a significant positive correlation with performance control sense (*r* = 0.41). Both psychological resilience and performance control sense were negatively associated with pre-performance rumination and MPA, with correlation coefficients ranging from −0.71 to −0.16. In contrast, pre-performance rumination was significantly and positively correlated with MPA (*r* = 0.47).

**Table 3:** Pearson correlations between variables.

**Variables**	**1**	**2**	**3**	**4**
Resilience	1			
Performance control sense	0.41^***^	1		
Pre-performance rumination	−0.16^***^	−0.54^***^	1	
MPA	−0.41^***^	−0.71^***^	0.47^***^	1

^***^p < 0.001.

MPA, music performance anxiety.

### 3.4 Mediation analyses

**Figure 2:**
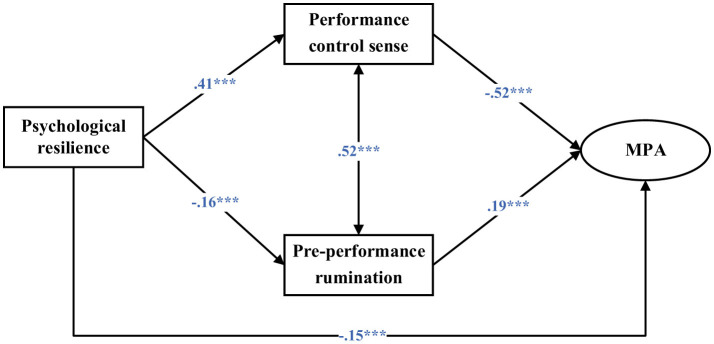
Structural equation modeling (MPA, music performance anxiety). Standardized coefficients were reported. ***, *p* < 0.001.

As illustrated in Figure 2, the mediation model demonstrated an acceptable overall fit to the data ($\chi_{\left(11\right)}^{2}$ = 112.55, CFI = 0.96, TLI = 0.91, SRMR = 0.06, RMSEA = 0.07). The direct effect of psychological resilience on MPA was statistically significant (β = −0.15, *p* < 0.001). In the first pathway, psychological resilience was positively associated with performance control sense (β = 0.41, *p* < 0.001), which was in turn negatively associated with MPA (β = −0.52, *p* < 0.001). This indicates that performance control sense partially mediated the relationship between psychological resilience and MPA, accounting for 53.8% of the total effect (*p* < 0.001). In the second pathway, psychological resilience was negatively associated with pre-performance rumination (β = −0.16, *p* < 0.001), which was in turn positively associated with MPA (β = 0.19, *p* < 0.001). Accordingly, pre-performance rumination also served as a partial mediator of the resilience–MPA association, explaining 7.7% of the total effect.

### 3.5 Multi-group SEM

**Figure 3:**
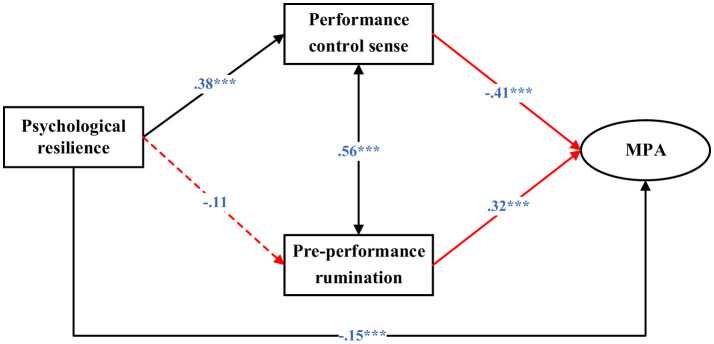
Structural equation modeling for males (MPA, Music performance anxiety). Standardized coefficients were reported. ***, *p* < 0.001.

**Figure 4:**
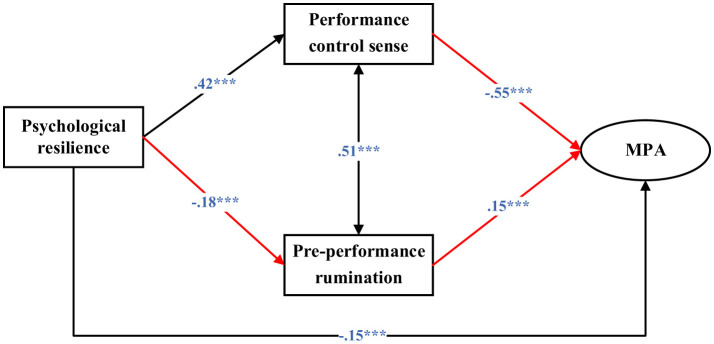
Structural equation modeling for females (MPA, Music performance anxiety). Standardized coefficients were reported. ***, *p* < 0.001.

As depicted in Figures 3, 4, the association between psychological resilience and pre-performance rumination was not statistically significant among males (β = −0.11, *p* = 0.110), whereas it was significant among females (β = −0.18, *p* < 0.001). The negative relationship between performance control sense and -MPA was stronger for females (β = −0.55, *p* < 0.001) compared with males (β = −0.41, *p* < 0.001). Conversely, the positive association between pre-performance rumination and MPA was more pronounced among males (β = 0.32, *p* < 0.001) than among females (β = 0.15, *p* < 0.001).

## 4 Discussion

The present study aimed to examine the direct and indirect associations between psychological resilience and MPA in a sample of Chinese choir members. Specifically, we tested the mediating roles of performance control sense and pre-performance rumination, as well as the potential sex differences. The findings generally supported our hypotheses (H1–H4), with both theoretical and practical implications for understanding and alleviating MPA in the context of collective musical performance.

### 4.1 The associations between demographic variables and MPA

The present findings revealed several demographic correlates of MPA. Age was negatively associated with MPA, suggesting that older participants experienced lower levels of anxiety, possibly due to accumulated performance experience and enhanced emotional regulation with age (Lupiáñez et al., 2021). Specifically, female participants reported higher levels of MPA than males, a result consistent with prior research indicating that female tend to experience greater performance-related anxiety across musical and non-musical contexts (Barros et al., 2024). Consistent with previous studies emphasizing the role of socioeconomic resources, higher self-reported household income was associated with lower MPA. Individuals with greater financial security may experience reduced stress and enhanced access to coping resources, such as private instruction or performance opportunities, which can buffer against anxiety (Musgrave et al., 2025). Similarly, participants with full-time employment reported less MPA than students or unemployed individuals, possibly reflecting greater life stability, broader performance experience, or higher self-efficacy acquired through occupational demands (Nedelcu et al., 2018). In contrast, educational attainment and partnership status were unrelated to MPA, indicating that general education and relational factors may exert limited influence on situational performance stress. Overall, these results underscore the importance of considering social and economic circumstances when examining MPA and suggest that financial and occupational stability may serve as protective factors against performance-related anxiety in community musicians.

### 4.2 Psychological Resilience as a protective factor against MPA

Consistent with H1, psychological resilience emerged as a robust negative predictor of MPA. This result aligns with the conceptualization of psychological resilience as a positive psychological quality that enables individuals to recover from stress and maintain adaptive functioning in the face of adversity (Lu et al., 2024b). For musicians, stage performance represents a highly evaluative and stressful context, often characterized by intense audience scrutiny and fear of negative evaluation (Papageorgi, 2020). Our findings reinforce the notion that resilient performers are better equipped to cope with these challenges and are therefore less likely to experience debilitating anxiety. Previous studies of classical musicians and music students have similarly identified psychological resilience as a protective factor against MPA (Kegelaers et al., 2021). The present results extend this evidence to Chinese choir members, a relatively under-studied population in the performance psychology literature. Importantly, choir singing differs from solo performance in its collective nature; members are simultaneously embedded in a group and evaluated as part of a collective artistic expression. This dual context may activate social evaluation concerns while also providing opportunities for mutual support and emotional buffering (Durrant, 2005; Welch et al., 2014). The significant negative association between psychological resilience and MPA suggests that resilient individuals are able to capitalize on these group dynamics, framing performance challenges as manageable and less threatening.

### 4.3 Performance control sense as a major mediator

Supporting H2, performance control sense was found to partially mediate the relationship between psychological resilience and MPA, accounting for more than half (53.8%) of the total effect. This is a particularly important finding, as it highlights the central role of control beliefs in performance contexts. Drawing on Social Cognitive Theory (Bandura, 2001), individuals with strong control beliefs are more likely to view performance situations as challenges that can be mastered rather than threats to be avoided. Such perceptions minimize anxiety by directing attention toward preparation and skill execution rather than potential failures. Recent work has also demonstrated that perceived control is negatively associated with maladaptive coping strategies and positively linked to mastery experiences (Frazier et al., 2011). The large mediation effect in the current study underscores the likelihood that psychological resilience exerts much of its protective function through its capacity to bolster performers' sense of control. From an applied perspective, these findings suggest that interventions aiming to reduce MPA should not only target resilience broadly but should specifically cultivate performance control sense. Cognitive-behavioral training, mastery rehearsal, and exposure to evaluative environments may strengthen performers' beliefs in their ability to manage performance situations (Burin and Osório, 2016). For choir members, structured rehearsal strategies and conductor feedback that emphasize mastery and competence may be particularly effective in enhancing perceived control, thereby reducing MPA.

### 4.4 Pre-performance rumination as a secondary mediator

In line with H3, pre-performance rumination also mediated the resilience–MPA relationship, though its contribution was comparatively modest (7.7% of the total effect). This finding provides support for Gross's process model of emotion regulation (Gross and Thompson, 2007), which highlights the importance of antecedent-focused strategies in shaping emotional outcomes. Resilient individuals are more likely to engage in adaptive strategies such as cognitive reappraisal, reducing the tendency to ruminate excessively about possible performance failures. The smaller mediation effect suggests that while rumination plays a role, it is less central than performance control in explaining how psychological resilience buffers against MPA. This result may reflect cultural nuances: in collectivist societies such as China, cognitive constructs related to control and mastery may be more salient determinants of MPA than internal ruminative tendencies (Li et al., 2024). Additionally, choir members perform within a collective setting, where shared responsibility may dilute the personal significance of rumination (Su et al., 2025).

Nonetheless, the role of rumination should not be overlooked. Consistent with previous research (Tanovic et al., 2017), excessive anticipatory rumination has been shown to exacerbate MPA by depleting attentional resources and reinforcing negative self-evaluations. Our findings indicate that interventions aimed at reducing MPA could benefit from incorporating mindfulness-based approaches, acceptance strategies, or metacognitive training to disrupt maladaptive ruminative cycles. Even if the overall mediation effect is modest, targeting rumination may still yield meaningful reductions in anxiety for specific subgroups of performers.

### 4.5 Sex differences

Consistent with H4, multi-group SEM revealed that some hypothesized pathways differed significantly by sex. First, psychological resilience was negatively associated with pre-performance rumination among females but not among males. This finding resonates with evidence that females tend to rely more heavily on ruminative coping (Johnson and Whisman, 2013). Psychological resilience may therefore play a particularly important role in mitigating females' susceptibility to rumination, serving as a stronger protective factor in this group (Ji, 2024). Second, the association between performance control sense and MPA was stronger among females compared to males. This result suggests that females' anxiety is more sensitive to fluctuations in perceived control. According to sex role theory (Eagly and Wood, 2016), females are socialized to be more attentive to social evaluation and interpersonal feedback, which may amplify the role of control beliefs in regulating anxiety. This also aligns with research indicating that female musicians often report heightened self-consciousness and sensitivity to evaluative threats (Cui et al., 2024). Third, the association between pre-performance rumination and MPA was stronger among males than females. This somewhat counterintuitive result may be explained by differences in coping congruence. While females frequently ruminate and may develop strategies to buffer its impact, rumination is less typical of males' coping repertoires (Lilly et al., 2023). Consequently, when males engage in rumination, it may be particularly maladaptive, leading to disproportionately high levels of anxiety (Espinosa et al., 2022). This interpretation echoes findings from stress and coping research showing that incongruence between typical coping styles and situational demands can exacerbate distress (McLean et al., 2011). Together, these results highlight the need for sex-sensitive approaches to understanding and addressing MPA. For females, strengthening control beliefs may be particularly effective, whereas for males, interventions targeting maladaptive rumination may hold greater promise.

### 4.6 Theoretical and practical contributions

This study provides both theoretical and practical contributions to the understanding of MPA. Theoretically, it extends the literature by integrating psychological resilience, performance control, and rumination into a comprehensive model, empirically validating their interrelations within a choir performance context. By examining sex differences, the study further demonstrates that the protective effects of psychological resilience and the mechanisms through which it operates are not uniform across sexes. This aligns with broader psychological theories emphasizing sex differences in emotional regulation and stress coping (Sardella et al., 2022). Moreover, the study refines existing conceptualizations of psychological resilience by showing that its effects are largely mediated through cognitive appraisals of control rather than solely through the suppression of maladaptive thought patterns such as rumination. This finding underscores the centrality of cognitive appraisals in MPA and contributes to ongoing debates about the primary mechanisms through which psychological resilience exerts its benefits.

Practically, the findings highlight several implications for music education and performance practice. Educators and conductors should consider implementing training programs that simultaneously build psychological resilience and strengthen control beliefs (Cheung et al., 2024). Structured rehearsal strategies, opportunities for mastery experiences, and constructive feedback may serve to enhance both psychological resilience and perceived control, while mindfulness and metacognitive training could help performers reduce maladaptive rumination (Osborne et al., 2014). Importantly, the observed sex differences suggest that interventions should be tailored: for female choir members, programs that emphasize self-efficacy, perceived control, and mastery may be particularly effective, whereas for male members, strategies that directly address ruminative tendencies, such as mindfulness, acceptance, and cognitive restructuring, may be more impactful. At a broader policy level, these findings underscore the value of integrating psychological resilience training into music education curricula in China. With the rapid expansion of art education and the increasing number of individuals engaged in choral singing, fostering psychological resources such as psychological resilience may play a critical role in promoting not only artistic success but also performers' mental health and wellbeing.

### 4.7 Limitations

Several limitations should be noted. First, the cross-sectional design precludes causal inference among psychological resilience, mediating variables, and MPA. Longitudinal research is warranted to examine the temporal dynamics of these relationships. Second, data were based on self-reports, which may be affected by social desirability bias. Third, participants were members of community choirs, who may experience lower evaluative pressure than professional or conservatory musicians. This sampling characteristic might have influenced the intensity and structure of MPA observed. Consistent with Spahn et al. (2023), performance context and expertise level are crucial in shaping MPA; therefore, future studies should replicate and extend the present model among professional and student musicians performing under higher performance demands to test the robustness of the mediating mechanisms identified.

## 5 Conclusions

This study shows that psychological resilience is a key protective factor against in Chinese choir members. Psychological resilience reduced MPA both directly and indirectly, mainly through enhancing performance control sense and, to a lesser degree, through lowering pre-performance rumination. Performance control accounted for the larger share of the effect, highlighting its central role in the resilience–MPA link. Notably, sex differences emerged: control beliefs were more protective for females, while rumination had a stronger adverse effect for males. These findings deepen theoretical understanding of the mechanisms connecting psychological resilience and MPA and emphasize the need for sex-sensitive interventions. Training that strengthens psychological resilience and control while reducing rumination may help cultivate healthier, more confident, and more resilient performers.

## Data Availability

The raw data supporting the conclusions of this article will be made available by the authors, without undue reservation.

## References

[B1] Albaqawi H. Alamri M. Al-Dossary R. Al Hosis K. Alharbi J. Aljohani M.. (2025). Exploring the impact of self-efficacy social support and learning environment on clinical performance anxiety in student nurses. Sci. Rep. 15:8663. 10.1038/s41598-025-93400-y 40082643 PMC11906794

[B2] Ayed M. Ejheisheh M. A. Ayed A. Batran A. (2025). Understanding the relationship between resilience and psychological well-being among nursing students in Palestine. BMC Nurs. 24:889. 10.1186/s12912-025-03566-z 40634916 PMC12239407

[B3] Baminiwatta A. Fernando R. Gadambanathan T. Jiyatha F. Maryam K. H. Premaratne I.. (2025). The buffering role of resilience on burnout, depression, anxiety, and stress among healthcare workers in Sri Lanka. Discov. Psychol. 5:19. 10.1007/s44202-025-00345-4

[B4] Bandura A. (2001). Social cognitive theory: an agentic perspective. Ann. Rev. Psychol. 52, 1–26. 10.1146/annurev.psych.52.1.1 11148297

[B5] Barros S. Marinho H. França A. Pereira A. (2024). Music performance anxiety: a study of anxiety predictors in higher education music students in Portugal. Int. J. Music Educ. 1–16. 10.1177/02557614241280040

[B6] Boardman J. D. Blalock C. L. Button T. M. M. (2008). Sex differences in the heritability of resilience. Twin Res. Hum. Genet. 11, 12–27. 10.1375/twin.11.1.12 18251671 PMC2674367

[B7] Booth J. W. Neill J. T. (2017). Coping strategies and the development of psychological resilience. J. Outdoor Environ. Educ. 20, 47–54. 10.1007/BF03401002

[B8] Burin A. B. Osório F. D. L. (2016). Interventions for music performance anxiety: results from a systematic literature review. Arch. Clin. Psychiatry 43, 116–131. 10.1590/0101-60830000000097

[B9] Chang X. Guo C. Zhou H. Liu L. (2023). Impact of rumination on sleep quality among patients with non-alcoholic fatty liver disease: a moderated mediation model of anxiety symptoms and resilience. BMC Psychiatry 23:84. 10.1186/s12888-023-04572-8 36732707 PMC9893673

[B10] Cheung A. T. Ho L. L. K. Li W. H. C. Chan G. C. F. Choi K. C. Chung J. O. K.. (2024). Group-based instrumental musical training to enhance resilience among school-aged children from low-income families: a pilot randomised waitlist controlled trial. Nurs. Open 11:e2134. 10.1002/nop2.2134 38481006 PMC10937816

[B11] Connor K. M. Davidson J. R. T. (2003). Development of a new resilience scale: the connor-davidson resilience scale (CD-RISC). Depress. Anxiety 18, 76–82. 10.1002/da.10113 12964174

[B12] Cui C. Xie X. Yin Y. (2024). Exploring the relationships among music performance anxiety, teaching anxiety, and self-efficacy of Chinese preservice music teachers. Front. Psychol. 15:1373454. 10.3389/fpsyg.2024.1373454 38680289 PMC11048473

[B13] Durrant C. (2005). Shaping identity through choral activity: singers‘ and conductors' perceptions. Res. Stud. Music Educ. 24, 88–98. 10.1177/1321103X050240010701

[B14] Eagly A. H. Wood W. (2016). “Social role theory of sex differences,” in The Wiley Blackwell Encyclopedia of Gender and Sexuality Studies, eds. A. Wong, M. Wickramasinghe, R. Hoogland, and N. A. Naples (Chichester: Wiley), 1–3. 10.1002/9781118663219.wbegss183

[B15] Espinosa F. Martin-Romero N. Sanchez-Lopez A. (2022). Repetitive negative thinking processes account for gender differences in depression and anxiety during adolescence. J. Cogn. Ther. 15, 115–133. 10.1007/s41811-022-00133-1 35251444 PMC8881790

[B16] Frazier P. Keenan N. Anders S. Perera S. Shallcross S. Hintz S. (2011). Perceived past, present, and future control and adjustment to stressful life events. J. Pers. Soc. Psychol. 100, 749–765. 10.1037/a0022405 21299308

[B17] Gómez-López B. Sánchez-Cabrero R. (2023). Current trends in music performance anxiety intervention. Behav. Sci. 13:720. 10.3390/bs13090720 37753998 PMC10525579

[B18] Gross J. J. Thompson R. A. (2007). “Emotion regulation: conceptual foundations,” in Handbook of Emotion Regulation (New York, NY, US: The Guilford Press), 3–24.

[B19] Guyon A. J. A. A. Hildebrandt H. Güsewell A. Horsch A. Nater U. M. Gomez P. (2022). How audience and general music performance anxiety affect classical music students' flow experience: a close look at its dimensions. Front. Psychol. 13:959190. 10.3389/fpsyg.2022.959190 36389478 PMC9649719

[B20] Ji L. (2024). Childhood emotional abuse and depression among Chinese adolescent sample: a mediating and moderating dual role model of rumination and resilience. Child Abuse Negl. 149:106607. 10.1016/j.chiabu.2023.106607 38154376

[B21] Johnson D. P. Whisman M. A. (2013). Gender differences in rumination: a meta-analysis. Pers. Individ. Diff. 55, 367–374. 10.1016/j.paid.2013.03.019 24089583 PMC3786159

[B22] Kaleńska-Rodzaj J. (2019). Pre-performance emotions and music performance anxiety beliefs in young musicians. Res. Stud. Music Educ. 42, 77–93. 10.1177/1321103X19830098

[B23] Kaleńska-Rodzaj J. (2021). Music performance anxiety and pre-performance emotions in the light of psychology of emotion and emotion regulation. Psychol. Music 49, 1758–1774. 10.1177/0305735620961154

[B24] Kegelaers J. Schuijer M. Oudejans R. R. D. (2021). Resilience and mental health issues in classical musicians: a preliminary study. Psychol. Music 49, 1273–1284. 10.1177/0305735620927789

[B25] Kenny D. T. (2009). “The factor structure of the revised Kenny music performance anxiety inventory,” in International Symposium on Performance Science (Utrecht: Association Européenne des Conservatoires), 37–41.

[B26] Kenny D. T. (2023). The Kenny music performance anxiety inventory (K-MPAI): scale construction, cross-cultural validation, theoretical underpinnings, and diagnostic and therapeutic utility. Front. Psychol. 14:1143359. 10.3389/fpsyg.2023.1143359 37325731 PMC10262052

[B27] Kinney C. Saville P. Heiderscheit A. Himmerich H. (2025). Therapeutic interventions for music performance anxiety: a systematic review and narrative synthesis. Behav. Sci. 15:138. 10.3390/bs15020138 40001769 PMC11851691

[B28] Kline R. B. (2016). Principles and Practice of Structural Equation Modeling, 4th Edn. New York, NY, US: The Guilford Press.

[B29] Leng H. Xiang X. Li S. (2025). The chain mediation effects of general self-efficacy and psychological resilience between physical activity and academic stress among Chinese adolescents. Front. Psychol. 16:1626157. 10.3389/fpsyg.2025.1626157 40823422 PMC12354399

[B30] Li J. Jiang X. Zhou Y. (2024). Culture, emotion, and cognition: understanding the psychological dynamics of Chinese sports with emotional regulation skills and cognitive reappraisal. Heliyon 10:e34306. 10.1016/j.heliyon.2024.e34306 39108858 PMC11301204

[B31] Li Y. Zheng P. (2025). Trait resilience protects against social anxiety in college students through emotion regulation and coping strategies. Sci. Rep. 15:28143. 10.1038/s41598-025-13674-0 40751070 PMC12317036

[B32] Lilly K. J. Howard C. Zubielevitch E. Sibley C. G. (2023). Thinking twice: examining gender differences in repetitive negative thinking across the adult lifespan. Front. Psychol. 14:1239112. 10.3389/fpsyg.2023.1239112 38022916 PMC10663279

[B33] Lima A. A. de Ximenes R. C. C. de Souza S. L. (2024). Factors associated with music performance anxiety in adolescents: a systematic review. Children Youth Serv. Rev. 164:107860. 10.1016/j.childyouth.2024.107860

[B34] Little T. D. (2013). Longitudinal Structural Equation Modeling. New York, NY, US: The Guilford Press.

[B35] Liu D. Wang Y. Xie P. Deng H. Qiu L. Liu W.. (2023). Rumination and depression in chinese adolescents with mood disorders: the mediating role of resilience. J. Clin. Psychiatry 84:22m14682. 10.4088/JCP.22m14682 37498649

[B36] Lu H. Xia S. Zheng Y. Chen W. Jin Z. Sun W.. (2025). The associations between coping resources and help-seeking intention in a sample of Chinese first-year medical students: mediation effects of coping strategies. BMC Public Health 25:1579. 10.1186/s12889-025-22755-8 40295998 PMC12036150

[B37] Lu H. Yang J. Zhao K. Jin Z. Wen X. Hu N.. (2024a). Perceived risk of COVID-19 hurts mental health: the mediating role of fear of COVID-19 and the moderating role of resilience. BMC Psychiatry 24:58. 10.1186/s12888-024-05511-x 38254008 PMC10802027

[B38] Lu H. Yu Y. Wang D. B. Wu A. M. S. Chen J. H. Zhang G.. (2024b). Association between interpersonal resources and mental health professional help-seeking among Chinese adolescents with probable depression: mediations via personal resources and active coping. BMC Psychiatry 24:840. 10.1186/s12888-024-06271-4 39574049 PMC11580335

[B39] Lupiáñez M. Ortiz F. de P. Vila J. Muñoz M. A. (2021). Predictors of music performance anxiety in conservatory students. Psychol. Music 50, 1005–1022. 10.1177/0305735621103229022983129

[B40] Maddux J. E. Kleiman E. M. (2021). “Self-efficacy: the power of believing you can,” in The Oxford Handbook of Positive Psychology, 3rd Edn (New York, NY, US: Oxford University Press), 443–452.

[B41] McLean C. P. Asnaani A. Litz B. T. Hofmann S. G. (2011). Gender differences in anxiety disorders: prevalence, course of illness, comorbidity and burden of illness. J. Psychiatr. Res. 45, 1027–1035. 10.1016/j.jpsychires.2011.03.006 21439576 PMC3135672

[B42] Moral-Bofill L. López de la Llave A. Pérez-Llantada M. C. Holgado-Tello F. P. (2022). Development of flow state self-regulation skills and coping with musical performance anxiety: design and evaluation of an electronically implemented psychological program. Front. Psychol. 13:899621. 10.3389/fpsyg.2022.899621 35783805 PMC9248863

[B43] Mulawarman M. Rindi Antika E. Afriwilda M. T. Prabawa A. F. Nadhita G. Purboaji N. (2024). How does resilience predict cognitive rumination in college students? Konselor 12, 302–312. 10.24036/0202312443-0-86

[B44] Musgrave G. Gross S. A. Carney D. (2025). Determinants of anxiety, depression and subjective wellbeing among musicians in denmark: findings from the ‘when music speaks' project. Scand. J. Psychol. 66, 429–445. 10.1111/sjop.13095 39930749 PMC12042730

[B45] Nedelcu S. Leucuta D. Dumitrascu D. (2018). Lifestyle and psychosocial factors in musicians. Clujul Med. 91, 312–316. 10.15386/cjmed-959 30093810 PMC6082614

[B46] Osborne M. S. Franklin J. (2002). Cognitive processes in music performance anxiety. Aust. J. Psychol. 54, 86–93. 10.1080/00049530210001706543

[B47] Osborne M. S. Greene D. J. Immel D. T. (2014). Managing performance anxiety and improving mental skills in conservatoire students through performance psychology training: a pilot study. Psychol. Well-Being 4:18. 10.1186/s13612-014-0018-3

[B48] Papageorgi I. (2020). Prevalence and predictors of music performance anxiety in adolescent learners: contributions of individual, task-related and environmental factors. Musicae Scientiae 26, 101–122. 10.1177/1029864920923128

[B49] Ping Chuang S. Yung Wei Wu J. Shu Wang C. (2024). Impact of rumination and psychological distress on the quality of sleep: a moderated role of resilience in community adults. Am. J. Health Behav. 48, 1557–1565. 10.5993/AJHB.48.6.7

[B50] Rudenstine S. Feliz S. Brown O. Schulder T. (2025). Defying risk: moving from resilience to the capacity to adapt, a contribution to mental health prevention. Front. Psychol. 16:1600841. 10.3389/fpsyg.2025.1600841 40672847 PMC12263666

[B51] Sardella A. Lenzo V. Basile G. Musetti A. Franceschini C. Quattropani M. C. (2022). Gender and psychosocial differences in psychological resilience among a community of older adults during the COVID-19 pandemic. J. Pers. Med. 12:1414. 10.3390/jpm12091414 36143198 PMC9504613

[B52] Spahn C. Tenbaum P. Immerz A. Hohagen J. Nusseck M. (2023). Dispositional and performance-specific music performance anxiety in young amateur musicians. Front. Psychol. 14:1208311. 10.3389/fpsyg.2023.1208311 37583605 PMC10425269

[B53] Stuart N. C. Zoccola P. M. Dickerson S. S. (2025). Diurnal cortisol and rumination: examining gender differences. Psychoneuroendocrinology 179:107517. 10.1016/j.psyneuen.2025.107517 40517526

[B54] Su P. Jiang K. Kong J. (2025). Flow in choral singing: associations with perceived choral memory performance and well-being among older adults. BMC Psychol. 13:892. 10.1186/s40359-025-03276-w 40790228 PMC12341315

[B55] Su Y.-H. Ho Y.-C. Cheng Y.-R. Chen H.-S. (2017). The development of “anticipatory music performance anxiety inventory” and “stage music performance anxiety inventory.” Psychol. Testing 64, 207–235. 10.7108/PT.201709_64(3).0002

[B56] Tanovic E. Hajcak G. Sanislow C. A. (2017). Rumination is associated with diminished performance monitoring. Emotion 17, 953–964. 10.1037/emo0000290 28252977 PMC6425483

[B57] Wang Q.-R. Yang R. (2024). The influence of music performance anxiety on career expectations of early musical career students: self-efficacy as a moderator. Front. Psychol. 15:1411944. 10.3389/fpsyg.2024.1411944 38915430 PMC11194429

[B58] Wang S. Li J. Zhao X. Zhou M. Zhang Y. Yu L.. (2023). Perceived stress mediates the association between perceived control and emotional distress: the moderating role of psychological resources and sex differences. J. Psychiatric Res. 168, 240–248. 10.1016/j.jpsychires.2023.10.051 37922598

[B59] Watkins E. R. Roberts H. (2020). Reflecting on rumination: consequences, causes, mechanisms and treatment of rumination. Behav. Res. Ther. 127:103573. 10.1016/j.brat.2020.103573 32087393

[B60] Welch G. F. Himonides E. Saunders J. Papageorgi I. Sarazin M. (2014). Singing and social inclusion. Front. Psychol. 5:803. 10.3389/fpsyg.2014.00803 25120514 PMC4114289

[B61] Yang Y. Lei P. Huang Z. Yu H. Zhang H. (2025). The impact of professional music performance competence on performance anxiety: the mediating role of psychological risk and moderating role of psychological resilience. Front. Psychol. 16:1565215. 10.3389/fpsyg.2025.1565215 40115283 PMC11924045

[B62] Yu Z. Liu W. (2024). The psychological resilience of teenagers in terms of their everyday emotional balance and the impact of emotion regulation strategies. Front. Psychol. 15:1381239. 10.3389/fpsyg.2024.1381239 40052017 PMC11883688

[B63] Zeng W. Wu X. Xu Y. Wu J. Zeng Y. Shao J.. (2021). The impact of general self-efficacy on psychological resilience during the COVID-19 Pandemic: the mediating role of posttraumatic growth and the moderating role of deliberate rumination. Front. Psychol. 12:684354. 10.3389/fpsyg.2021.684354 34248788 PMC8261126

[B64] Zhang H. Jenatabadi H. S. (2024). Effects of social support on music performance anxiety among university music students: chain mediation of emotional intelligence and self-efficacy. Front. Psychol. 15:1389681. 10.3389/fpsyg.2024.1389681 39377059 PMC11457729

